# Disparities in Dental Service Utilization among Adults in Chinese Megacities: Do Health Insurance and City of Residence Matter?

**DOI:** 10.3390/ijerph17186851

**Published:** 2020-09-19

**Authors:** Xiaomin Qu, Xiang Qi, Bei Wu

**Affiliations:** 1 School of Social Development, East China University of Political Science and Law, 555 Longyuan Road Songjiang District, Shanghai 201620, China; millie_qu@163.com; 2 Rory Meyer College of Nursing, New York University, 433 First Avenue, New York, NY 10010, USA; xq450@nyu.edu; 3 Rory Meyer College of Nursing, NYU Aging Incubator, New York University, 433 First Avenue, New York, NY 10010, USA

**Keywords:** Chinese, dental visits, health insurance, city of residence, health disparities

## Abstract

The aims of the study were to present the prevalence of dental service utilization among adults (age between 18 and 65) in Chinese megacities and to examine the associations of health insurance and city of residence with dental visits. This study was a cross-sectional analysis of the 2019 New Era and Living Conditions in Megacities Survey data with a sample of 4835 participants aged 18–65 from 10 different megacities in China. The data including gross domestic product (GDP) per capita of each megacity obtained from the National Bureau of Statistics of China as a city-level characteristic. After adjusting sampling weights, approximately 24.28% of the participants had at least one dental visit per year. Findings from multilevel mixed-effects linear models showed that participants residing in megacities with higher GDP per capita (β = 0.07, *p* < 0.001) who had Urban Employee Basic Medical Insurance (β = 0.25, *p* < 0.001) or Urban Resident Basic Medical Insurance (β = 0.19, *p* < 0.01) had more frequent dental visits after adjusting demographic characteristics, socioeconomic status, health status, health behavior and attitude, and oral health indicators. Margins post-estimation model results demonstrated disparities in the predicted probability of having never visited a dentist by types of health insurance and city of residence. In conclusion, the prevalence of dental visits in China was found to be low. This study highlights socioeconomic inequalities in dental service utilization. There is a great need to develop more dental care programs and services and expand health insurance to cover dental care in China.

## 1. Introduction

Oral diseases are global public health issues because of their prevalence and impact on individuals and societies [1]. Approximately 3.5 billion people worldwide live with oral health diseases and problems, according to the Global Burden of Disease 2015 report [2]. Oral health status is associated with physical function, cognitive function, and many chronic diseases such as diabetes and cardiovascular diseases [3,4,5,6,7]. Oral diseases and problems are widespread in China [8]. Data from the 4th National Oral Health Epidemiological Survey showed that 87.4% of middle-aged adults aged 35–44 were affected by gum bleeding [9]. Regular dental visits are considered a major determinant of oral health and its related quality of life [10,11,12,13,14]. Unequal access to dental care services may lead to inequalities in oral health outcomes [15]. Disparity in dental service utilization is a global challenge [16] and its relationship with socioeconomic status has been well documented in both high-income and low-income countries (e.g., US, European countries, Canada, Japan, Sweden, Finland, Indonesia, Iran, Chile) [15,17,18,19,20,21,22,23,24]. However, very few studies have reported on dental service utilization in China, and even fewer studies have examined disparities in dental service utilization.

### 1.1. Dental Service Utilization: Oral Health Care Challenges in China

Despite the very high prevalence of oral health problems [9], dental care service utilization is low in China [8,25,26]. The prevalence of dental care visits among adults aged 45 and above was 18.1% in 2015 [25,26]. Although China has undergone a dramatic social transition and achieved significant economic development, its dental care delivery system is underdeveloped, and the dental resources are unevenly distributed, particularly between urban and rural areas [8]. Additionally, dental care literacy among the Chinese population is still far behind developed countries [27]. Most Chinese adults do not seek dental services unless they experience tooth pain or other oral health problems [28]. All of these challenges highlighted the urgent need for policies, practice, and studies in addressing disparities in dental service utilization.

### 1.2. Health Insurance: Unequal Access to Dental Care Services

The Chinese government implemented a new round of health system reforms in 2009 and established multiple health insurance plans determined by occupation and place of residence (i.e., urban or rural) [29]. Currently, there are three types of health insurance plans [30] among urban residents: Government Medical Insurance (GMI, established in 1988), Urban Employee Basic Medical Insurance (UEBMI, established in 1998), and Urban Resident Basic Medical Insurance (URBMI, established in 2007). Only government employees and retirees are eligible to benefit from the GMI. The UEBMI covers urban employees and retirees. As a voluntary insurance, the URBMI covers children, students, seniors, and unemployed residents. Participants of the URBMI need to pay premium contributions every year (nearly 200 RMB, equivalent to 30 dollars) [31,32]. In the rural areas, there was no health insurance plan until the New Cooperative Medical Scheme (NCMS) was established in 2009 [30]. Compared to other health insurance plans in urban areas, the NCMS is for rural residents, and it covers only minimal medical expenses and does not cover dental care costs [30]. Megacities comprise not only urban residents but also rural residents. However, the proportion of the urban population varies greatly across different megacities. For example, approximately 87% of the population were urban residents in Beijing in 2018 [33], while the proportion of urban residents was only approximately 49% in Chongqing the same year [34].

Health insurance coverage and copayment can partially contribute to the disparities in dental visits [35,36]. In China, different health insurance plans reflect individuals’ socioeconomic positions, affordability, and access to healthcare and dental care resources. Different from the NCMS, the three other health insurance plans for urban residents cover quite a few treatment-oriented dental care costs. Additionally, the reimbursement rate, premium contributions, and deductibles vary strongly among health insurance plans. In general, the health care and drug packages covered by the UEBMI are more generous than those of the URBMI and the NCMS. The cost reimbursement rate is about 90–95% for the GMI, 60–75% for the UEBMI, and 50–65% for the UEBMI [30]. Most preventative oral health services in China are utilized among financially well-off individuals [25], because prevention-oriented dental care services are not covered by any insurance mentioned above [37]. Compared to participants covered by the UEBMI, rural residents covered by the NCMS have lower incomes and unstable occupation, and thus less ability to pay for dental care services.

### 1.3. City of Residence: Unequal Delivery of Dental Care Services

The utilization of dental care services may also vary across places of residence because geographic location affects individuals’ access to health care services [38]. Despite China’s rapid economic development, the level of economic development varies significantly from region to region, and there is a large variation in availability of health care resources, including dental care services [39]. The level of oral health literacy also varies across cities in China. Residents in more developed areas may have better dental care knowledge. Moreover, health insurance plans are managed by city-level local government in China; the details regarding the utilization and reimbursement of health insurance policies vary based on regional interpretation of national laws [29]. In this case, examining the association between the city of residence and regular dental visits helps to understand the regional inequality of dental service utilization in China.

### 1.4. Current Study

Dental care utilization in China is at a deficient level, and the majority of dental visits are not prevention-oriented. So far, very few studies have examined disparities in dental care in China. One study we found was focused on urban–rural differences [8]. Studies on health care utilization in China suggested regional inequalities in the distribution of healthcare resources [38,39]. Dental care resources are also unevenly distributed across cities. However, few studies emphasized health insurance and city of residence as important socioeconomic indicators of dental service utilization. Identifying contributing factors of inequality is important for informing equity-oriented programs and policies. Findings may provide insights in other countries that are also making efforts to promote access to dental care. 

This present study aimed to examine the associations between health insurance, city of residence, and dental visits among adults in Chinese megacities. Two hypotheses were proposed: (1) participants covered by certain health insurance plans (UEBMI and URBMI) have more frequent dental visits than those with other health insurance or without any health insurance; and (2) in comparison to those in megacities with lower gross domestic product (GDP) per capita, participants residing in megacities with higher GDP per capita have more frequent dental visits.

## 2. Methods

### 2.1. Data and Sample

The individual-level characteristics, such as frequency of dental visits, health insurance, demographic characteristics, socioeconomic status, health status, health behavior and attitude, and oral health status of participants in this study were obtained from the 2019 New Era and Living Conditions in Megacities Survey (NELCMS), a cross-sectional survey focusing on policy issues such as social structure and social mobility. While the city-level data, GDP per capita, was obtained from the website of the National Bureau of Statistics of China [40].

The 2019 NELCMS was conducted in 10 megacities in China, including the most economically developed megacities with GDP per capita higher than 134,000 RMB (equivalent to 19,619 US dollars), namely Beijing, Shanghai, Guangzhou, Shenzhen, and other megacities, such as Tianjin, Hangzhou, Chongqing, Chengdu, Wuhan, and Changsha. A multistage stratified sampling strategy was used. Participants were selected in four stages: city, neighborhood, household, and individual. Forty neighborhoods were randomly selected from each megacity. Twenty-five households were randomly selected from the housing registration database obtained from each neighborhood community. One individual was randomly selected from each household. Data were collected during in-home interviews by well-trained interviewers. Inclusion criteria for participation in the survey were: (1) full-time residence in this city for more than six months in the past year; (2) age between 18 and 65; (3) capable of answering interview questions. In this survey, questionnaires included Volume A and Volume B. Only Volume B included the oral health-related questions, resulting in an actual sample of 5000 participants for this study. After excluding 149 participants with missing values and 16 participants without any teeth (edentulism), the final analytical sample consisted of 4835 individuals. We did not include individuals with complete tooth loss because the dental care utilization pattern is very different between those with and without natural teeth [41]. 

### 2.2. Measures

#### 2.2.1. Dental Visits

Dental visits were measured by the question: “How often do you visit a dentist for dental care?” The responses were grouped into six categories: “never” = 0, “rarely” = 1, “less than once every two years” = 2, “at least once every two years” = 3, “at least once a year” = 4, or “twice a year” = 5.

#### 2.2.2. Health Insurance and City of Residence

As an individual-level independent variable, types of health insurance were coded as five dummy variables: covered by the NCMS (0 = no, 1 = yes), the URBMI (0 = no, 1 = yes), the UEMBI (0 = no, 1 = yes), the GMI (0 = no, 1 = yes), other medical insurance (including commercial medical insurance, 0 = no, 1 = yes). No health insurance coverage was treated as the reference.

As a city-level independent variable, GDP per capita was used as a proxy of the characteristics of city of residence. Among the 10 megacities, Shenzhen had the highest GDP per capita (nearly 189,568 RMB, equivalent to 27,755 US dollars), while Chongqing had the lowest (nearly 63,689 RMB, equivalent to 9325 US dollars) [40]. 

#### 2.2.3. Covariates

We included demographic characteristics, socioeconomic status, health status, health behavior and attitude, and oral health status as covariates; (i) demographic characteristics included gender (0 = female, 1 = male), actual age (in years), marital status (0 = otherwise, 1 = married or living with partner); (ii) socioeconomic status included education (in years of education completed, ranging from 0–22 years), income (categorized into four groups: I, II, III, IV, according to the first quartile, mean, and the third quartile); (iii) health status included self-rated health measured by nine questions acquired from the Self-rated Health Measurement Scale (SRHMS) [42]. Each item was scored on a scale of 0–10, with higher values representing greater self-rated health. The potential range of the scale was 0–90. The standardized Cronbach’s β-coefficient alpha for the measure was 0.8383; (iv) health behavior and attitude included regular physical exam (having physical exam once a year, 0 = no, 1 = yes), and care about healthy food (0 = no, 1 = yes); (v) oral health status included self-rated oral health (1 = very poor, 2 = poor, 3 = fair, 4 = good, 5 = very good), tooth loss (the number of missing teeth), and gum bleeding (1 = never, 2 = hardly, 3 = occasionally, 4 = frequently, 5 = always). 

### 2.3. Statistical Analysis

**Figure 1:**
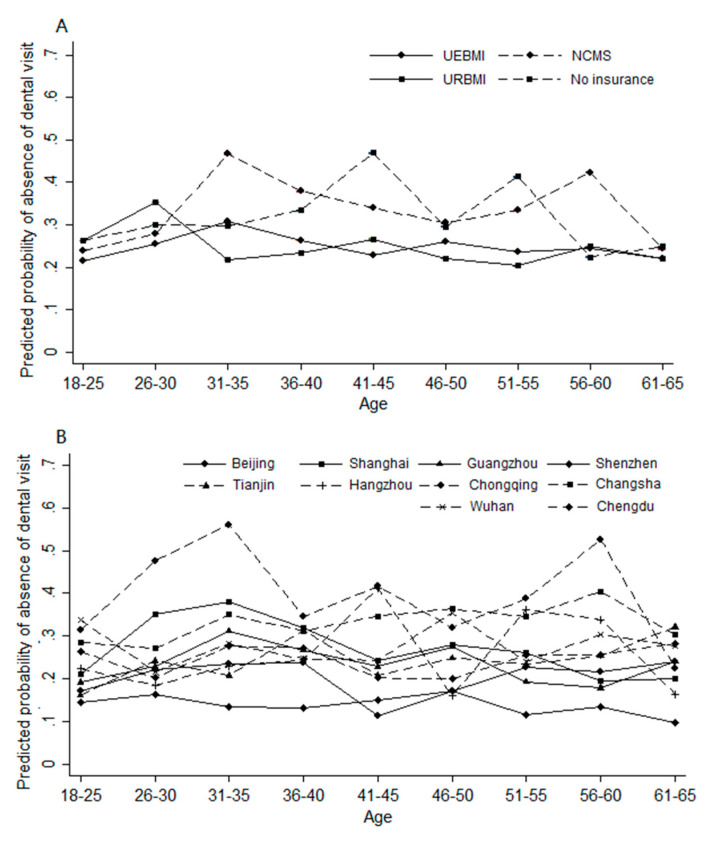
Disparities in predicted probability of absence of dental visit over age (weighted). (**A**) Predicted probability of absence of dental visit over age by health insurance with 95% Cls. (**B**) Predicted probability of absence of dental visit over age by city of residence with 95% Cls. Notes: absence of dental visit indicates having never visited a dentist; all estimations include gender, age, marital status, education years, income, self-rated health, regular physical exam, care about healthy food, self-rated oral health, tooth loss, and gum bleeding as covariates.

We used Stata 15 for all statistical analyses [43]. Since this study was designed to estimate the frequency of dental visits and its associated factors, adjustment of variances to reflect the multi-stage stratified sampling was essential [44]. Individual-level sampling weights were applied to reflect the complex sampling design in the 2019 NELCMS. We defined city as SU (*n* = 10), and neighborhood as strata (*n* = 40). The analysis presented in Tables 1 and 2 and Figure 1 adjusted for weighting [45]. 

Descriptive analysis was first conducted in both unweighted and weighted samples. Weighted frequency of dental visits was calculated, and Chi-square tests were applied to compare differences between groups. Multilevel mixed-effects linear models were applied to examine the associations between health insurance, city of residence (GDP per capita), and dental visits. Then, margins post-estimation models were applied to evaluate whether the predicted probability of having never visited a dentist differed by health insurance or city of residence [46,47]. 

## 3. Results

### 3.1. Descriptive Statistics

Descriptive characteristics of both unweighted and weighted analytical samples are presented in Table 1. After adjusting sampling weights, approximately 90% of the participants had health insurance coverage. Specifically, 11.12% of participants were covered by the NCMS, 24.11% by the URBMI, 45.77% by the UEBMI, 2.71% by the GMI, and 6.67% by other medical insurance (including commercial insurance). In the city-level characteristics, the mean of the GDP per capita among these 10 megacities was nearly 129,300 RMB (equivalent to 18,931 US dollars). 

### 3.2. Health Insurance Coverage and City of Residence by Dental Visits

Table 2 shows that, after adjusting sampling weights, only 24.28% of participants had at least one dental visit per year. Chi-squared tests showed significant differences in dental visits across different types of health insurance and city of residence. Participants who had more frequent dental visits tended to be covered by the UEBMI as opposed to other health insurance or without any health insurance (*p* < 0.001). In addition, participants who resided in specific megacities, such as Beijing and Shenzhen, had more dental visits than those who resided in other megacities (*p* < 0.001). For example, 35.61% of participants who resided in Beijing and 36.03% who resided in Shenzhen had at least one dental visit per year. 

### 3.3. Multilevel Mixed-Effects Linear Models 

Table 3 presents the multilevel mixed-effects linear models for factors associated with dental visits. Adjusted for demographic characteristics and socioeconomic status, Model 2 shows that a higher GDP per capita was associated with more dental visits (β = 0.08, *p* < 0.001). After adding health insurance variables to the model, Model 3 shows that participants who had the UEBMI (β = 0.43, *p* < 0.001) and the URBMI (β = 0.30, *p* < 0.01) had significantly more frequent dental visit than those with other health insurance or without any health insurance. Model 4 shows that having a regular physical exam (β = 0.61, *p* < 0.001) and caring about healthy food (β = 0.28, *p* < 0.001) were associated with more frequent dental visits. Model 5 shows that better self-rated oral health was related to a lower frequency of dental visits. In contrast, oral health indicators, such as more tooth loss and a higher prevalence of gum bleeding, were associated with more frequent dental visits. 

### 3.4. Disparities in Dental Visits

Figure 1 shows the predicted probability of having never visited a dentist over age by health insurance status and city of residence after control for covariates. As shown in Figure 1A, participants covered by the UEBMI and the URBMI had a lower predicted probability of having never visited a dentist than those covered by the NCMS or without any health insurance coverage in different age groups. Similarly, participants who resided in megacities with higher GDP per capita, Beijing and Shenzhen in particular, had a lower predicted probability of having never visited a dentist than those who resided in other megacities (Figure 1B).

## 4. Discussion

This study presented the prevalence of dental visits and examined the associations of health insurance and city of residence with dental visits in ten megacities of China. Overall, only 24.28% of the participants had at least one dental visit per year. Participants covered by specific health insurance programs (UEBMI and URBMI) with higher reimbursement rates and who resided in megacities with higher GDP per capita had more dental visits after adjusting key covariates. Both hypotheses were supported.

Previous studies indicated a low prevalence of dental visits in China, especially among rural areas [8,25,26]. This study is in agreement with the previous findings. Even in the most economically developed megacities, such as Beijing and Shenzhen, only approximately 36.00% of the participants had at least one dental visit per year, which is much lower than 66.2%, the prevalence of dental service utilization among adults aged over 30 in the United States [17].

Findings from multilevel mixed-effects models and margins post-estimation models showed that the frequency of dental visits was associated with the type of health insurance. Although China has made efforts to expand health insurance coverage, the NCMS was not available for rural residents until 2009 [30]. The NCMS covers very limited medical expenses and dental care costs. Thus, dental care utilization of participants with the NCMS remains extremely low. Approximately 48.00% of participants with the NCMS had never visited a dentist, and only 11.63% of them had at least one dental visit per year. In comparison to urban residents with other health insurance plans, rural residents have more limited affordability and access to dental care services.

Even the UEBMI and the URBMI for urban residents only cover basic treatment-oriented dental services and do not include consultation, dental check-up, or any prevention-oriented dental care services. Therefore, over 85% of the dental costs have to be paid out of pocket [37]. In this way, the ability to pay is closely related to dental care utilization. Obviously, more efforts should be made to provide a universal health insurance that covers dental care expenses. A reform to integrate different health insurance plans could be one of the key strategies in addressing inequalities in dental visits in China. In recent years, the Chinese government has made efforts to narrow the gap between different population groups in terms of health insurance. Some local governments have taken measures to combine the UEBMI, URBMI, and GMI into the Uniform Social Basic Medical Insurance [48] in order to improve residents’ access to health care services. Given the low dental service utilization of participants covered by the NCMS, more efforts are needed to target rural areas.

This study also found that participants who resided in more economically developed megacities were more likely to have more frequent dental visits. A possible explanation is that higher socioeconomic development in an area is closely related to not only better dental care service delivery, but also higher dental care literacy among residents. Another possible explanation is that levels of health care resources, including dental care resources, and health insurance policies vary across cities [29,39]. To address regional disparities in dental visits, local governments should take action to improve dental care service delivery and access to health care resources, including dental care services.

It is interesting that in this study, poor oral health outcomes were associated with more dental visits. These findings reflect the findings that the purpose of most dental visits in China are for dental treatments, and not for preventive dental check-ups. Dental check-ups are not common even in the most economically developed megacities in China. Chinese adults do not visit dentists until they encounter severe oral health problems [28]. For many low income and rural residents in China, in most cases, they avoid seeking dental care even if they have severe dental problems, such as tooth pain [49]. Pain killers are commonly used to treat tooth pain. In addition, when individuals have tooth pain, they are more likely to attribute it to Vitamin C insufficiency and “internal fire” (i.e., excessive body heat). Thus, they turn to Vitamin C supplements and drinking tea for help rather than visiting dentists [27]. The dental health knowledge of the Chinese population also needs to be improved. Having regular physical exams and caring about healthy food are significantly related to regular dental visits within the health behavior and attitude domain. It is likely that individuals who have more healthy behaviors and more positive attitudes will have better health knowledge, pay more attention to their oral health, and seek dental care services more consciously.

As a strategic policy aimed at improving health equity, the Healthy China 2030 Plan was released by the Chinese government in 2016. This blueprint described the challenges of oral health problems in China, and mid-term and long-term oral health promotion in the near future [50]. The plan emphasized the importance of implementing primary prevention efforts against oral health problems, integrating oral health into the promotion of overall health, and managing oral health disease in conjunction with other chronic diseases with shared risk factors. Further efforts should be made to promote the utilization of dental care services. Exploring the social determinants of dental service utilization will contribute to a better understanding of oral health inequalities in China.

Among the few studies that focus on disparities in dental service utilization in China, to the best of our knowledge, this is the latest study based on large-scale representative social survey data. Given that very few large-scale surveys in China include information on oral health status and dental service utilization, the availability of the 2019 New Era and Living Conditions in Megacities Survey data offered us the opportunity to better understand dental service utilization inequalities in Chinese megacities. This study has extended previous research by examining social determinants of dental service utilization inequalities beyond the urban–rural disparities [8].

There are some limitations to this study. First, given the nature of the cross-sectional study, we were unable to make causal inferences between dental care utilization and its associated factors. Second, although it covered ten megacities in China, our study is not based on a nationally representative sample. Third, some key variables, such as oral health behaviors and oral health literacy, are not available in this dataset. However, known explanatory factors like socioeconomic status, self-reported oral health outcomes, and education level were included. These variables ensured the explanatory power of this study. Fourth, we lack the information on the purpose of dental visits (e.g., regular check-ups and restorative procedures). Fifth, we lack medical and dental records of the participants in the survey. Health status and oral health status were self-reported, and we were not able to validate the self-reported data against clinical examined records. More efforts should be made to link social survey data and medical and dental records in future studies. In addition, further studies are needed to examine the trend of more specific dental service utilization in China.

## 5. Conclusions

In conclusion, this study found that the prevalence of dental visits in China is low, even in the most developed megacities. In addition, this study demonstrates that health insurance type and city of residence (GDP per capita) are associated with dental visits among adults in Chinese megacities. This finding may contribute to the understanding of dental service utilization inequalities. To promote dental service utilization and reduce oral health inequity in China, it is imperative to develop more dental care programs and services and expand health insurance to cover dental care.

## Figures and Tables

**Table 1:** Sample characteristics (*n* = 4835, unweighted and weighted).

Variables	Unweighted Sample		Weighted Sample	
	*n* (%)	M (SD)	*n* (%)	M (SD)
**Individual-level characteristics**				
**Gender**				
Male	2206 (45.63%)		2217 (45.85%)	
Female	2629 (54.37%)		2618 (54.15%)	
**Age**				
(18–25)	530 (10.96%)		514 (10.62%)	
(26–30)	507 (10.49%)		495 (10.24%)	
(31–35)	558 (11.54%)		543 (11.23%)	
(36–40)	510 (10.55%)		488 (10.10%)	
(41–45)	476 (9.84%)		474 (9.80%)	
(46–50)	530 (10.96%)		555 (11.48%)	
(51–55)	440 (9.10%)		454 (9.39%)	
(56–60)	493 (10.20%)		507 (10.48%)	
(61–65)	791 (16.36%)		805 (16.65%)	
**Marital status**				
Married or living with partner	3657 (75.64%)		3677 (76.05%)	
Otherwise	1178 (24.36%)		1158 (23.95%)	
**Education years**		12.56 (3.78)		12.41 (3.81)
**Income**				
I	1267 (26.20%)		1274 (26.34%)	
II	1347 (27.86%)		1392 (28.78%)	
III	1266 (26.18%)		1273 (26.33%)	
IV	955 (19.75%)		897 (18.54%)	
**Self-rated health** (SRMHS scores)		61.77 (12.87)		61.69 (12.81)
**Regular physical exam**				
Yes	2962 (61.26%)		2891 (59.79%)	
No	1873 (38.74%)		1944 (40.21%)	
**Care about healthy food**				
Yes	2822 (58.37%)		2739 (56.65%)	
No	2013 (41.63%)		2096 (43.35%)	
**Self-rated oral health**				
Very poor	147 (3.04%)		140 (2.90%)	
Poor	908 (18.78%)		910 (18.82%)	
Fair	1223 (25.29%)		1173 (24.25%)	
Good	2045 (42.30%)		2056 (42.52%)	
Very good	512 (10.59%)		556 (11.50%)	
**Tooth loss**		1.38 (2.82)		1.36 (2.83)
**Gum bleeding**				
Never	978 (20.23%)		969 (20.03%)	
Hardly	1445 (29.89%)		1465 (30.31%)	
Occasionally	1953 (40.39%)		1955 (40.44%)	
Frequently	399 (8.25%)		390 (8.07%)	
Always	60 (1.24%)		56 (1.15%)	
**Health insurance coverage**				
No health insurance	472 (9.76%)		465 (9.61%)	
New Cooperative Medical Scheme	514 (10.63%)		538 (11.12%)	
Urban Resident Basic Medical Insurance	1155 (23.89%)		1166 (24.11%)	
Urban Employee Basic Medical Insurance	2263 (46.80%)		2213 (45.77%)	
Government medical insurance	133 (2.75%)		131 (2.71%)	
Other medical insurance	298 (6.16%)		323 (6.67%)	
**City-level characteristics**				
GDP per capita (10,000 RMB)		12.93 (3.35)		12.24 (3.55)

Notes: *n* = number; M = mean; SD = standard deviation; GDP = gross domestic product.

**Table 2:** Health insurance coverage and city of residence by dental visits (weighted).

Variables	Total	Frequency of Dental Visits *n* (%)						*p*-Value
		Never	Rarely	Less Than Once Every Two Years	At Least Once Every Two Years	At Least Once a Year	Twice a Year	
**Total**	N = 4835	1443 (29.84%)	1601 (33.11%)	239 (4.94%)	379 (7.83%)	891 (18.43%)	283 (5.85%)	
**Health insurance coverage**								
No health insurance	465 (9.61%)	190 (40.78%)	155 (33.38%)	25 (5.43%)	27 (5.73%)	48 (10.28%)	20 (4.39%)	<0.001
New Cooperative Medical Scheme	538 (11.12%)	260 (48.25%)	166 (30.92%)	17 (3.23%)	32 (5.96%)	45 (8.34%)	18 (3.29%)	
Urban Resident Basic Medical Insurance	1166 (24.11%)	333 (28.61%)	429 (36.80%)	57 (4.93%)	79 (6.79%)	203 (17.40%)	64 (5.48%)	
Urban Employee Basic Medical Insurance	2213 (45.77%)	557 (25.19%)	665 (30.03%)	118 (5.34%)	202 (9.14%)	513 (23.19%)	157 (7.10%)	
Government medical insurance	131 (2.71%)	38 (28.68%)	56 (40.64%)	6 (4.41%)	6 (4.22%)	16 (12.19%)	10 (7.85%)	
Other medical insurance	323 (6.67%)	66 (20.22%)	130 (40.26%)	15 (4.62%)	33 (10.20%)	66 (20.53%)	13 (4.16%)	
**City of residence**								
Beijing	629 (13.01%)	125 (19.88%)	215 (34.16%)	18 (2.90%)	47 (7.45%)	171 (27.12%)	53 (8.49%)	<0.001
Changsha	236 (4.87%)	106 (45.12%)	51 (21.75%)	11 (4.88%)	11 (4.67%)	41 (17.28%)	15 (6.30%)	
Chengdu	479 (9.91%)	151 (31.55%)	152 (31.75%)	30 (6.19%)	25 (5.15%)	97 (20.21%)	25 (5.15%)	
Guangzhou	422 (8.73%)	117 (27.63%)	143 (33.77%)	20 (4.82%)	29 (6.80%)	75 (17.76%)	39 (9.21%)	
Hangzhou	284 (5.87%)	74 (26.10%)	84 (29.65%)	9 (3.34%)	24 (8.56%)	60 (21.09%)	32 (11.27%)	
Shanghai	712 (14.73%)	182 (25.51%)	273 (38.27%)	53 (7.41%)	57 (8.02%)	114 (16.05%)	34 (4.73%)	
Shenzhen	389 (8.05%)	35 (8.91%)	46 (11.94%)	44 (11.34%)	124 (31.78%)	130 (33.40%)	10 (2.63%)	
Tianjin	451 (9.32%)	107 (23.64%)	226 (50.21%)	8 (1.88%)	10 (2.30%)	72 (15.90%)	27 (6.07%)	
Wuhan	325 (6.73%)	104 (31.98%)	115 (35.22%)	18 (5.67%)	14 (4.45%)	52 (15.99%)	22 (6.68%)	
Chongqing	907 (18.77%)	443 (48.77%)	296 (32.58%)	26 (2.87%)	37 (4.10%)	80 (8.81%)	26 (2.87%)	

Notes: *n* = number; comparisons between groups were done using the Chi-square test.

**Table 3:** Multilevel mixed-effects linear models for factors associated with dental visits (*n* = 4835).

	Model 1	Model 2	Model 3	Model 4	Model 5
	(Null model)	β (95%CI)	β (95%CI)	β (95%CI)	β (95%CI)
**Random effects**					
City-level variance (SE)	0.22 (0.10)	0.03 (0.02)	0.02 (0.01)	0.02 (0.01)	0.02 (0.01)
**Fixed effects**					
**City-level characteristics**					
GDP per capita (10,000 RMB)		0.08 (0.05, 0.12) ***	0.09 (0.05, 0.12) ***	0.08 (0.05, 0.11) ***	0.07 (0.04, 0.10) ***
**Individual-level characteristics**					
**Covariates**		√	√	√	√
**Health insurance coverage**					
No insurance			Ref	Ref	Ref
New Cooperative Medical Scheme			0.08 (−0.12, 0.28)	0.01 (−0.19, 0.20)	0.01 (−0.18, 0.21)
Urban Resident Basic Medical Insurance			0.30 (0.13, 0.47) **	0.18 (0.01, 0.35) *	0.19 (0.03, 0.35) *
Urban Employee Basic Medical Insurance			0.43 (0.27, 0.60) ***	0.26 (0.10, 0.43) **	0.25 (0.09, 0.41) **
Government medical insurance			−0.07 (−0.38, 0.24)	−0.21 (−0.51, 0.10)	−0.20 (−0.49, 0.10)
Other medical insurance			0.15 (−0.09, 0.39)	−0.03 (−0.26, 0.21)	0.02 (−0.21, 0.24)
**Self-rated health**					
SRHMS scores				−0.01 (−0.01, −0.01) ***	0.00 (0.00, 0.00)
**Regular physical exam**					
No				Ref	Ref
Yes				0.61 (0.51, 0.71) ***	0.60 (0.50, 0.70) ***
**Care about healthy food**					
No				Ref	Ref
Yes				0.28 (0.19, 0.38) ***	0.29 (0.20, 0.38) ***
**Self-rated oral health**					
Very poor					Ref
Poor					−0.15 (−0.42, 0.12)
Fair					−0.42 (−0.70, −0.15) **
Good					−0.64 (−0.92, −0.37) ***
Very good					−0.91 (−1.22, −0.60) ***
**Tooth loss**					
Number of missing teeth					0.04 (0.02, 0.05) ***
**Gum Bleeding**					
Never					Ref
Hardly					0.44 (0.21, 0.46) ***
Occasionally					0.55 (0.43, 0.67) ***
Frequently					0.62 (0.44, 0.81) ***
Always					0.70 (0.30, 1.10) **
**Log restricted-likelihood**	−9235.58	−9082.3339	−9059.2979	−8944.5676	−8795.3696
***p*-value**	0.000	0.000	0.000	0.000	0.000

Notes: All estimations include gender, age, marital status, education years, and income as covariates; SE = standard error; 95% confidence intervals in parentheses; *** *p* < 0.001, ** *p* < 0.01, * *p* < 0.05.
